# Effectiveness of supported housing versus residential care in severe mental illness: a multicenter, quasi-experimental study

**DOI:** 10.1007/s00127-021-02214-6

**Published:** 2022-01-18

**Authors:** Lorenz B. Dehn, Thomas Beblo, Dirk Richter, Günther Wienberg, Georg Kremer, Ingmar Steinhart, Martin Driessen

**Affiliations:** 1 Department of Psychiatry and Psychotherapy, Evangelisches Klinikum Bethel, University Hospital OWL, Remterweg 69-71, 33617 Bielefeld, Germany; 2 Center for Psychiatric Rehabilitation, Bern University Hospital for Mental Health, Bern, Switzerland; 3 Department of Psychiatry and Psychotherapy, University of Bern, Bern, Switzerland; 4 Department of Health Professions, Bern University of Applied Sciences, Bern, Switzerland; 5 Bethel.regional, Stiftung Bethel, Bielefeld, Germany; 6 von Bodelschwinghsche Stiftungen Bethel, Bielefeld, Germany; 7 Institut für Sozialpsychiatrie Mecklenburg-Vorpommern e. V., University of Greifswald, Greifswald, Germany

**Keywords:** Psychiatric rehabilitation, Health services, Supported accommodation, Social functioning

## Abstract

**Purpose:**

Up to now there are only few studies and no RCT comparing efficacy or effectiveness of supported housing (SH) versus residential care (RC) in severe mental illness (SMI) without homelessness. Here we present an observational follow-up study in SMI subjects, who entered SH or RC, to compare clinical and functional outcomes 2 years later.

**Methods:**

In this prospective study in more than 30 locations throughout a German federal state, we included SMI subjects, who entered SH (*n *= 153) or RC (*n *= 104). About one quarter suffered from each substance use, psychotic, affective, or other disorders. To avoid sampling bias, we used the propensity score matching method to establish a quasi-experimental design. Outcome measures were social functioning (SFS), the number of psychiatric hospitalisations, psychopathology (SCL-9-K), and quality of life (MANSA). Apart from descriptive methods we analysed primarily using repeated-measures ANOVAS.

**Results:**

Our analyses revealed significant effects of time for all outcomes in both study groups. However, there were not any group differences of outcome measures, i.e., not any significant effects of group or interactional effects of group x time. Moreover, these results hold true for intent-to-treat and per-protocol sample analyses.

**Conclusion:**

The results show, that SH and RC for non-homeless people with SMI achieve the same clinical and psychosocial outcomes across a 2-year period. Taking into account the users’ preferences, the present findings should give reason to ensure the availability of affordable housing and to support the expansion of supported housing approaches.

**Supplementary Information:**

The online version contains supplementary material available at 10.1007/s00127-021-02214-6.

## Introduction

Different forms of supported accommodation services for persons with a severe mental illness (SMI) have been established as a regular standard of psychiatric care since the deinstitutionalization processes of the last century [1]. Along the residential support continuum the most prominent types in the western world are residential care (RC) with more or less 24 h presence of a supporting staff and supported housing forms (SH), i.e., living in a normal flat with getting support by a staff several times per week, sometimes added by a back-up on-call service support up to 24 h 7 days a week. Despite the wide spread of these different housing services for non-homeless people with SMI in recent decades, there is still an urgent need of research on how efficious and effective RC and SH are in terms of social functioning, quality of life, psychopathology and hospitalisation. However, two consecutive Cochrane reviews concerning this issue were not able to identify any study (i.e., randomised controlled trial, RCT) that met the inclusion criteria [2, 3]. That is why Chilvers et al. [3] concluded that whether or not the benefits ('safe haven' with stability and support) of dedicated interventions associated with location in a building and with assistance from professional workers outweigh the risks (increasing dependence on professionals and prolonging exclusion from the community) “*can only be a matter of opinion in the absence of reliable evidence*” (p. 2)*.* MacPherson et al. [4] intended to compare 24-h residential care after discharge from psychiatric hospital with ongoing hospital care in subjects with schizophrenia but could only identify one small study (*n *= 22) which showed major limitations and received a poor methodological quality rating. In their systematic review, Richter and Hoffmann [5] found only eight studies in non-homeless mentally ill without any RCT. Outcome measures in terms of social integration, health status, and subjective evaluations did not yield in clear results in favour of SH versus RC. Costs were not investigated as an outcome measure in either of these eight studies. The authors conclude that “*there is still a lack of high-quality studies on non-homeless people that could inform social policy and funding bodies in countries where the main problem is not homelessness but the allocation of resources to the most effective and efficient housing settings*” (p. 7). Similarly, McPherson et al. [1] summarized their recent systematic review of mental health supported accommodation services by indicating a clear need for high quality effectiveness research.

That psychiatric supported housing is still under-researched may be due to different personal and structural reasons. For instance, Killaspy et al. [6] intended to perform a clinical trial comparing the effectivenes of supported housing versus floating outreach approach, but they were finally only successful in recruiting 8 participants who agreed to randomisation (and 9 others who agreed to participate in a naturalistic group) after screening 1432 persons. The authors reported major reasons not to participate in the trial were the staffs’ as well as service users’ preferences for one type of support. Staffs also felt that randomisation compromised their professional judgement. A further reason may be the legal claim that people with severe handicaps have in a variety of countries. For instance, in Germany, RCTs are difficult to perform, because persons have a right of social support including supported housing. The final decision, which kind of support is needed and financed, is most often done by the state social welfare institutions on the basis of a structured assessment (e.g., the Integrated Treatment and Rehabilitation Plan [7]) as well as evaluation of the subjects’ wishes.

### Aims of the study

To contribute more systematic research evidence, our study aimed to compare supported accommodation under RC versus SH conditions in a prospective 2-year study setting. Because of the availability and intensity of social, nursing, and therapeutic support (e.g., 24-h presence of staff members in RC versus 2 to 12 h per week in SH), we hypothesized that RC is more effective than SH in terms of psychopathology, hospitalisation, social functioning, and quality of life outcomes.

## Method

### Study design and setting

This is a multicenter observational 2-year follow-up study in persons with SMI, which started after entering RC or SH. We were not able to perform randomisation because of the above mentioned reasons. Thus we performed analyses using the propensity scoring approach [8]. Here we mainly present results of the intention-to-treat (ITT) sample but results of the per-protocol sample are also available as supplemental material.

The recruitment of study participants has involved four welfare organisations (v. Bodelschwinghsche Stiftungen Bethel, Bielefeld; Das Dach, Detmold; Förderkreis Sozialpsychiatrie, Münster; Wohnverbund Landschaftsverband Westfalen-Lippe, Münster), whose residential support services are spread throughout the Westfalian part of North-Rhine-Westfalia, Germany.

As reviews have repeatedly pointed out [1, 3, 5], there is a large variation in the supported accommodation services internationally due to different social legal frameworks and health care structures. To overcome the terminological inconsistency associated with this, the Simple Taxonomy for Supported Accommodation (STAX-SA [9]) has been developed as an overarching classification system. Following this STAX-SA-system, the RC settings in our study correspond to the supported accommodation types 1 and 2 with (more or less 24 h) staff on-site, high support, a congregate setting, and partly limited, but mostly strong emphasis on move-on. The SH settings match the STAX-SA type 4, where service users are visited by support staff in their individual, permanent tenancy, receive low to moderate (in some cases also high) support, with only limited emphasis on move-on. In the literature, this type of supported housing is sometimes also described as “floating outreach”. The differences in the STAX-SA characteristics described for our two types of supported accommodation are also reflected in the results of a novel housing fidelity scale for German-speaking countries that was recently developed and tested for the first time [10]. These results, which are also based on data from the present study’s supported accommodation services, show, that SH demonstrated higher fidelity compared to RC, especially for the domains of “Housing conditions” and “Inclusion orientation” [10].

### Procedures

Subjects with severe mental illness, who were intended to enter RC or SH or had entered one of these programs, were screened for inclusion and exclusion criteria based on the medical documentation. Inclusion criteria were as follows: (A) age 18 to 69 years old, (B.1) a severe mental illness, diagnosed by a psychiatrist which (B.2) lasted at least 2 years and (B.3) was associated with a handicap which constitutes the right for supported housing according to the German social law IX. The decision is based on a structured assessment provided by the staff of social welfare organisations or of a psychiatric hospital. Exclusion criteria were (A) non-sufficient German language capacities and (B) comorbid severe medical conditions (to avoid a confounding of functional and participation impairments caused by both somatic and psychiatric factors).

If the subjects were eligible to participate in the study, research workers visited the potential participants, verified the inclusion criteria, provided extended oral as well as written information, and obtained the written informed consent. Subsequently, the next possible baseline assessment (t1) was appointed within a time interval of maximum 6 weeks after RC/SH starting. An intermediate assessment was provided 1 year later (t2) and the final one 2 years later (t3). The participants received a compensation of 10 Euros for the first and 20 Euros for each of the two following assessments. The study was approved by the IRB (University of Muenster Ethics Committee, 2017-149-f-S).

### Measures

In accordance with the bio-psycho-social framework of the International Classification of Functioning, Disability and Health (ICF), we chose the following multidimensional outcome parameters: the primary outcome was social functioning as a measure of inclusion; in addition, the number of admissions to a psychiatric hospital, psychopathological symptom severity, and quality of life were used as secondary outcomes. All these measures were also described as “the most common” ones within a wide variety of possible mental health and psychosocial outcomes in the McPherson et al. [1] systematic review on supported accommodation services.

Sociodemographic and clinical characteristics (e.g., the occurrence of psychiatric admissions in the last 12 months) were obtained by a structured interview and the clinical diagnosis using the medical information provided by staff members and the administrative IT data system used by the institutions.

Social functioning, as the primary outcome, was assessed using the German version of the Social Functioning Scale (SFS) [11, 12]. The 76-items SFS covers a variety of aspects of social functions by 7 subscales (e.g., interpersonal behaviour, pro-social activities, independence, employment/occupation) and its sound psychometric characteristics have been repeatedly confirmed in different psychiatric samples [13, 14]. Here we used the standardised value of the total score.

The burden of psychopathology was assessed by the 9-item Symptom Checklist (SCL-K-9), a questionnaire, which was shown to have sufficient psychometric properties [15, 16]. Here we report the Global Severity Index (GSI).

Quality of life was assessed by the Manchester Short Assessment of Quality of Life (MANSA) ([17]; German version by Röpcke and Linau, unpublished). The questionnaire covers the subject’s general satisfaction as well as that regarding 16 life domains and is reported to have sufficient psychometric properties in people with mental illness [18].

### Sample

**Fig. 1:**
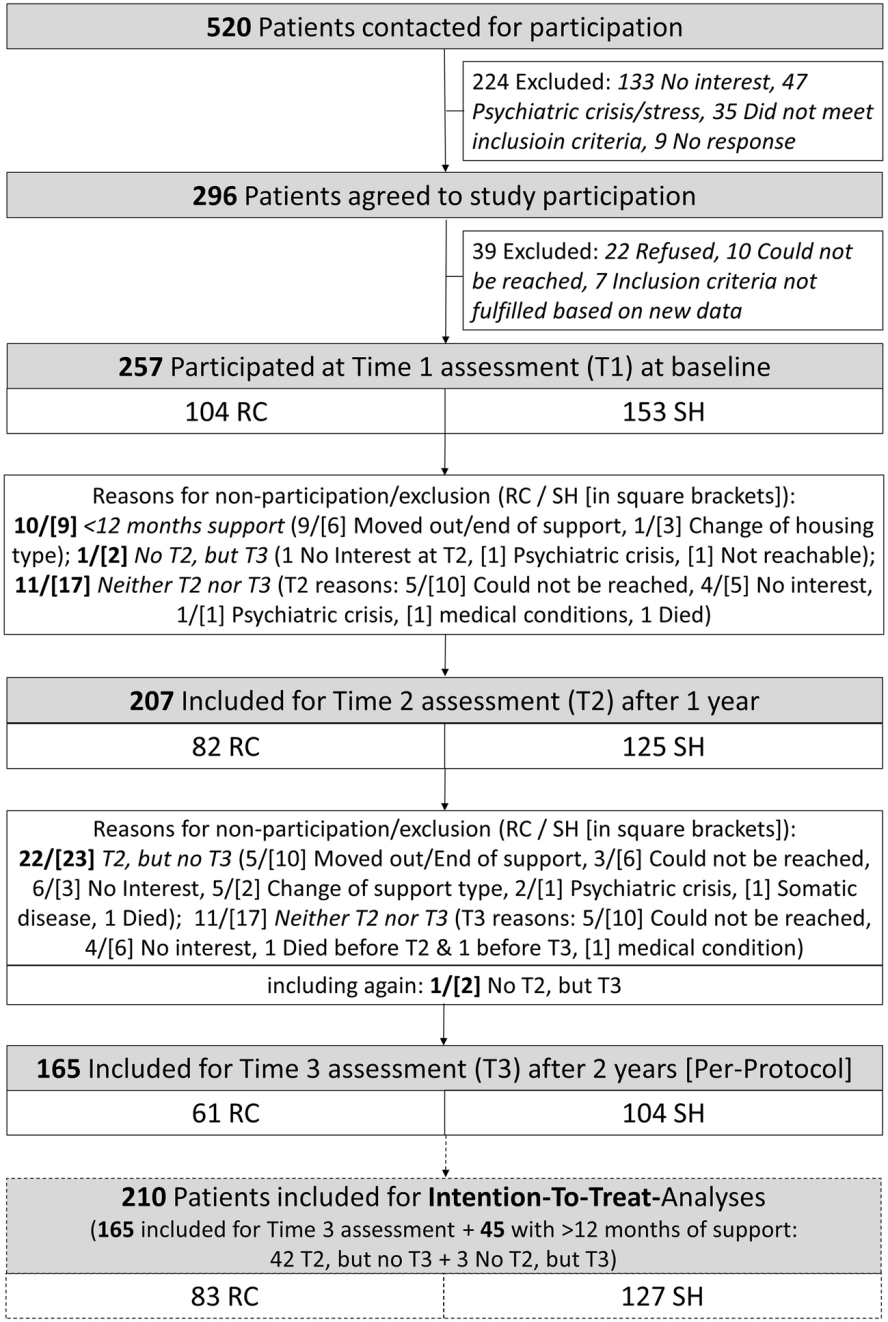
Study flowchart with patients from Residential Care (RC) and Supported Housing (SH)

A total of *n *= 520 subjects, who were just entering or had entered RC or SH (or who changed the housing type, accordingly) during the last 6 weeks, underwent the screening procedure (see Fig. 1). Of these, *n *= 296 agreed to participate and were included into the study. However, *n *= 36 persons had to be excluded for various reasons (see Fig. 1), so that the baseline assessment (t1) could finally be performed with *n *= 257 participants. Over the course of the study, there were *n *= 165 participants who completed the full 2 years in RC or SH and therefore constitute the per-protocol sample (RC *n *= 61, SH *n *= 104) with results being reported in the supplemental material. Another *n *= 45 participants fulfilled the minimum study requirement, i.e., they completed at least 1 year of the intervention. These *n *= 45 persons, together with the per-protocol group (*n *= 165), were defined to constitute the final ITT sample with a total of *n *= 210 participants (RC *n *= 83, SH *n *= 127). This ITT sample did not show significant differences in sociodemographic (age: *p* = 0.977, gender: *p* = 0.518) and psychosocial baseline-variables (SFS: *p* = 0.490, SCL: *p* = 0.981, MANSA: *p* = 0.706, ICD-diagnosis: *p* = 0.366) compared to the *n *= 47 drop-out persons from the initial study sample (*n *= 257).

Demographic and clinical characteristics of the ITT sample are reported in Table 1 for all participants and for the two major study groups RC and SH. The year before entering RC or SH nearly half of the participants (*n *= 102, 48.6%) had an/their own apartment, 30.9% changed within the psychiatric housing care system (from RC to SH or vice versa), 12% lived in changing or somewhat unclear forms of accommodation (sometimes with homelessness), and 8.5% came from other institutionalized accommodation settings (e.g., prison or youth center).

**Table 1:** Demographic and clinical characteristics of the total and the propensity score matched intention to-treat (ITT) sample at baseline [M (SD), *n* (%)]

	ITT sample				Propensity score matched ITT sample			
	RC *n *= 83	SH *n *= 127	Total *n *= 210	Statistic (RC vs. SH)	RC *n *= 62	SH *n *= 62	Total *n *= 124	Statistic (RC vs. SH)
Sex (female)	24 (28.9)	63 (49.6)	87 (41.4)	*Chi*^2^(1) = 8.85, ***p* = .004**, *V* = .205	19 (30.6)	22 (35.5)	41 (33.1)	*Chi*^2^(1) = 0.33, *p* = .703, *V* = .051
Age	40.4 (13.9)	40.6 (12.9)	40.5 (13.3)	*t*(208) = − 0.09, *p* = .928, *d* = 0.01	43.6 (14.3)	41.4 (12.5)	42.5 (13.4)	*t*(122) = 0.91, *p* = .366, *d* = 0.16
ICD Diagnosis								
F1	34 (41.0)	20 (15.7)	54 (25.7)	*Chi*^2^(4) = 46.06, ***p* < .001**, *V* = .468	24 (38.7)	20 (32.3)	44 (35.5)	*Chi*^2^(4) = 1.73, *p* = .806, *V* = .118
F2	29 (34.9)	16 (12.6)	45 (21.4)		19 (30.6)	16 (25.8)	35 (28.2)	
F3	7 (8.4)	41 (32.3)	48 (22.9)		7 (11.3)	10 (16.1)	17 (13.7)	
F4	5 (6.0)	17 (13.4)	22 (10.5)		4 (6.5)	5 (8.1)	9 (7.3)	
Other	8 (9.6)	33 (26.0)	41 (19.5)		8 (12.9)	11 (17.7)	19 (15.3)	
Prevalence of psychiatric admissions (last 12 months)	59 (71.1)	57 (44.9)	116 (55.2)	*Chi*^2^(1) = 13.94, ***p* < .001**, *V* = .258	43 (69.4)	37 (59.7)	80 (64.5)	*Chi*^2^(1) = 1.27, *p* = .348, *V* = .101
SCL-9-K	12.3 (8.1)	14.7 (8.7)	13.8 (8.5)	*t*(208) = − 2.02, ***p* = .045**, *d* = 0.25	11.5 (8.1)	13.1 (8.5)	12.3 (8.3)	*t*(122) = − 1.05, *p* = .295, *d* = 0.25
SFS total	105.1 (8.8)	104.9 (8.4)	104.9 (8.5)	*t*(208) = 0.19, *p* = .849, *d* = 0.02	104.8 (9.2)	104.9 (8.4)	104.8 (8.8)	*t*(122) = − 0.04, *p* = .964, *d* = 0.01
MANSA	37.4 (11.1)	36.9 (15.4)	37.1 (13.9)	*t*(206.1) = 0.29*p* = .773, *d* = 0.04	38.0 (11.8)	38.5 (15.7)	38.2 (13.8)	*t*(113.4) = − .23, *p* = .815, *d* = 0.04

Bold: significant difference at *p* < 0.05

*d* Cohens *d*, *V* Cramers V

The study baseline-assessments took place in 36 cities in Westphalia and included 39 separate RC homes as well as 50 separate SH-services by the 4 participating welfare organisations. The data collection ended in May 2020.

### Statistics

Baseline descriptive data and sample characteristics were assessed using Chi^2^- and T-tests. The main results were computed using two-way repeated measures analyses of variances (rm-ANOVA) to examine group-differences and progress of the two housing support groups on all numerical outcome variables. Between factor was group (RC versus SH) and within factor was time (baseline versus t3). The hypothesized advantage of the RC setting was tested by the interaction of these two factors, and Bonferroni-adjusted post hoc-tests (*α* = 0.025) were performed to further specify possible interaction effects. McNemar's test was used to compare repeated nominal data of RC and SH across the two time points.

#### Missing values handling

For the *n *= 45 participants of the ITT sample, who completed at least 1 year of the intervention but dropped out before the final t3 assessment (see flow chart, Fig. 1), we applied the Last Observation Carried Forward (LOCF) missing values handling approach to provide a conservative estimate of the t3 end-point data. For this, we considered the t2 assessment data as the last valid data point to be carried forward. The missing values for outcome variables in the ITT-sample (*n *= 210) were then imputed using the maximum likelihood-based expectation maximization (EM) procedure [19]. The proportion of missing data on outcome variables ranged from 2.9% (SCL-K9 at time 1) to 6.2% (MANSA at time 3). According to Little’s missing completely at random (MCAR)-test, it could be assumed that missing outcome data were completely at random, Chi^2^(168) = 187.94, *p* = 0.143. Despite its wide application of LOCF in clinical trials, however, statistical research has cautioned against the use of this missing data approach [20]. Therefore, we repeated all analyses using the EM procedure [21, 22] for missing numerical outcome data, including the missing values at the t3 study endpoint. Again, all missing outcome data were completely at random, according to Little’s MCAR test [Chi^2^(101) = 117.26, *p* = 0.128].

#### Matching procedure

Propensity score matching (PSM) was applied to balance the following important baseline covariates between the two groups: age, gender, ICD-10 diagnosis group, SCL-K9 score, and psychiatric hospital admissions during the 12 months before study entry. The PSM algorithm was based on logistic regression, a one-to-one matching method, without replacement, and with a caliper distance of 0.15 [23]. Using this method, a total of 62 pairs (*n *= 124 persons) were successfully matched and formed the propensity-score-matched ITT sample (see Table 1).

All statistical analyses were performed using the Statistical Software for the Social Sciences (SPSS Version 25). The general significance level was set to 0.05 and two-tailed. Appropriate effect sizes (Cramers-V, Cohens d) were provided for all analyses [24]. To strengthen the rigor of our statistical approach, we repeated all analyses using the PSM per-protocol-sample (*n *= 102) and the unmatched (naturalistic) ITT sample (*n *= 210). These additional results can be found in the supplemental material.

## Results

In the naturalistic ITT sample (without propensity-score matching, PSM), the two study groups were comparable at study entry with respect to age, social functioning (SFS) and quality of life (MANSA) (see Table 1). However, they significantly differed in terms of some demographic and clinical characteristics, i.e., a higher proportion of substance use disorders (ICD-10 F1) and schizophrenia (F2) in the RC group, and vice versa a higher proportion of affective (F3) and anxiety (F4) or other disorders in the SH group. In contrast, there was no longer a single significant difference between the study groups in the PSM ITT sample. This finding holds true both for the PSM ITT sample with LOCF- (Table 1) as well as with EM approach (Table S1).

**Fig. 2:**
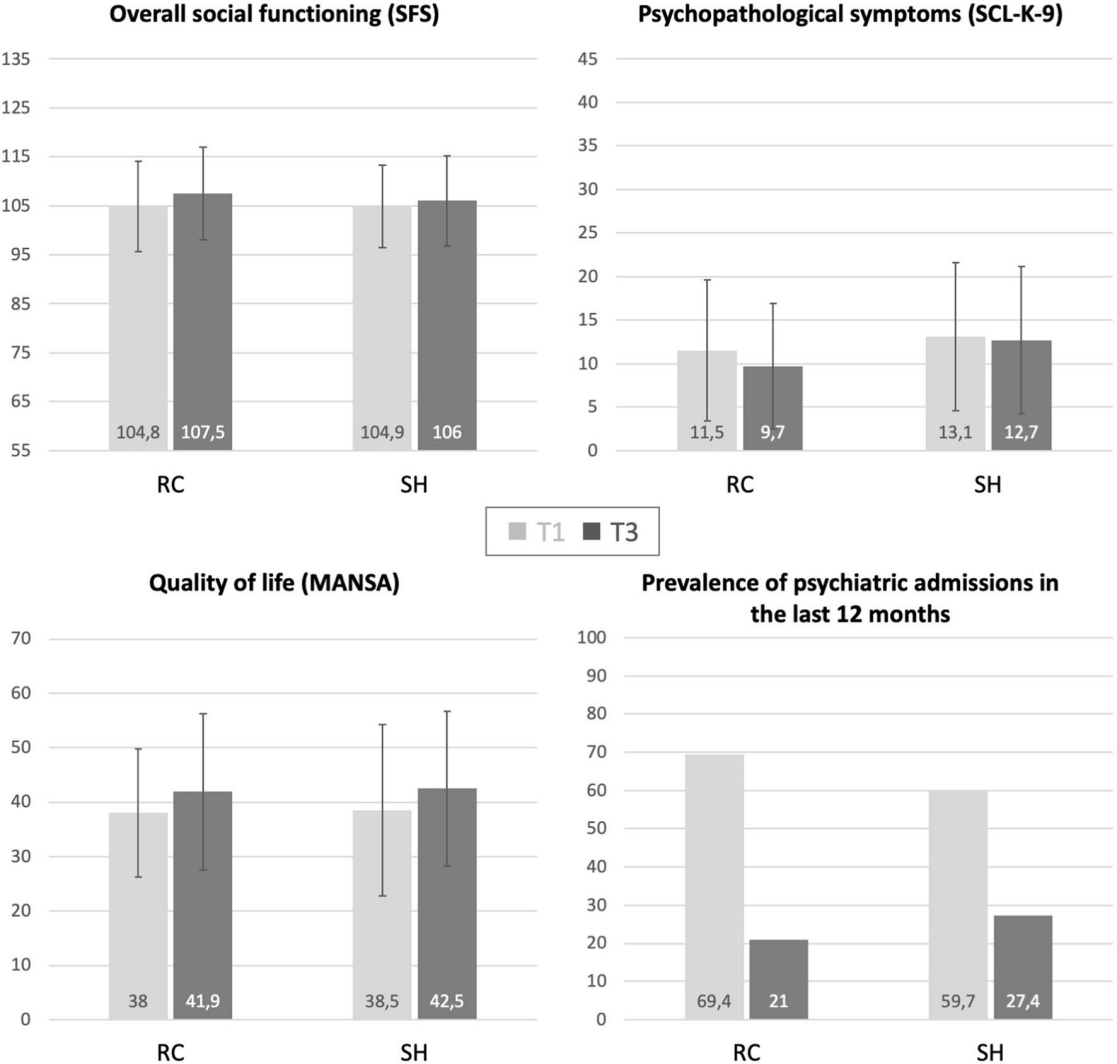
Descriptive results of the numerical (M, SD) and dichotomous (%) outcome measures for persons from Residential Care (RC) and Supported Housing (SH)

For the SFS total score, rm-ANOVA did not reveal any significant time x group interaction effect (see Table 2). While there was also no main effect of group (*p* = 0.630), the results yielded a significant main effect of time (*p* = 0.001). This indicates that both groups increased their overall social functioning across the study period, but we did not find any difference of effects between RC and SH. Similar results were also found regarding the other outcome measures with a significant increase of quality of life (MANSA, *p* < 0.001) and a non-significant reduction of psychopathological symptoms (SCL-K-9, *p* = 0.056) across the study period for both groups (see Fig. 2). The analyses using the PSM ITT sample with EM approach revealed the comparable findings (see Table 2) and also showed a significant decrease of psychopathology (SCL-K-9, *p *= 0.021).

**Table 2:** Results for the repeated-measures comparison of numeric (ANOVA) and dichotomous (McNemar) outcome variables

Outcome	PSM ITT sample^a^ (*LOCF*-method^b^)			PSM ITT sample^a^ (*EM-*method^c^)		
	Effects of time	Effects of group	Interaction time × group	Effects of time	Effects of group	Interaction time × group
Social Functioning (SFS total)	*F *= 10.88, ***p* = .001**, *d *= 0.60	*F *= 0.23, *p* = .630, *d *= 0.09	*F *= 1.93, *p* = .167, *d *= 0.26	*F *= 13.54, ***p* < .001**, *d *= 0.67	*F *= 0.51, *p* = .475, *d *= 0.13	*F *= 1.85, *p* = .176, ES = .24
Symptom level (SCL-K9)	*F *= 3.73, *p* = *.056*, *d *= 0.35	*F *= 3.00, *p* = .086, *d *= 0.31	*F *= 1.60, *p* = .208, *d *= 0.23	*F *= 5.47, ***p* = .021**, *d *= 0.42	*F *= 3.25, *p* = .074, *d *= 0.33	*F *= 2.03, *p* = .156, *d *= 0.25
Quality of Life (MANSA)	*F *= 15.27, ***p* < .001**, *d *= 0.71	*F *= 0.07, *p* = .793, *d *= 0.06	*F *< 0.01, *p* = .981, *d *< 0.06	*F *= 28.67, ***p* < .001**, *d *= 0.97	*F *= 0.83, *p* = .774, *d *= 0.06	*F *= 0.84, *p* = .361, *d *= 0.17
Prevalence of psychiatric admissions in the last 12 months^d^	McNemar: RC: *p* < .001, V = .170 SH: *p* < .001, V = .358		Chi^2^-test: t1: *p* = .348 t3: *p* = .530			

Bold: significant difference at *p* < 0.05

dF = 1 in outcome variables, error df = 122

^a^*PSM ITT* Propensity Score Matched intention to treat sample

^b^*LOCF* Last Observation Carried Forward

^c^*EM* maximum likelihood-based Expectation Maximization

^d^Dichotomous variables are not intended for the EM missing values method

The prevalence of psychiatric admissions in the last 12 months significantly decreased from t1 to t3 (see Table 2) both in the RC (69.4% to 21.0%, *p *< 0.001, V = 0.170) and the SH group (59.7% to 27.4%, *p *< 0.001, V = 0.358). The two groups did not differ in their prevalence of psychiatric admissions in the last 12 months at t1 (*p *= 0.348, see Table 1) or t3 (Chi^2^(1) = 0.70, *p *= 0.530, V = 0.075) (Fig. 2).

The aforementioned results were confirmed by the additional analysis of the propensity score matched per-protocol sample (*n *= 102, see Table S1), both using the LOCF- and the EM-missing values handling approach: the rm-ANOVAs again revealed only significant effects of time for all numerical outcome measures (*p *< 0.001 to *p *= 0.015), but not any significant group or interaction effects (see Table S2). Furthermore, we again found a statistically significant reduction of psychiatric hospitalisations for both the RC and the SH group, but no statistically significant difference at t3 (see Table S3). Furthermore, the results were also supported by the analysis of the unmatched (naturalistic) ITT sample (*n *= 210, see Tables S4-S5). However, the RC and SH subgroups were not comparable in some of their baseline values (compare with Table 1). Nevertheless, while there appeared only an additional significant group effect for the psychopathological symptoms (Table S4), all above mentioned results were confirmed again.

## Discussion

Our 2-year follow-up study revealed encouraging outcomes of Supported Housing (SH) as well as Residential Care (RC) in non-homeless people with severe mental illness, but we did not find any substantial differences between the two housing settings. This finding was backed up by differentiated statistical analyses and was shown for various clinical and psychosocial outcome parameters, i.e., social functioning, quality of life, psychopathology, and the number of psychiatric hospitalisations. These results are in agreement with the recent systematic review that also revealed no clear trends or at least similar outcomes, respectively, when comparing SH against RC regarding social integration, health status, and subjective evaluation outcome domains [5]. Moreover, as this systematic review revealed only two publications on non-homeless people from outside United States or Canada (i.e., Taiwan and Germany), our study provides empirical data to cover this research gap. The only reported study from Europe (Germany [25]) could indeed consider a large sample (*n *= 244) from different residential care settings, but all were located in a defined area around one larger capital city and all participants had a (chronic) schizophrenia. In contrast, the present study sample was collected (i) in a variety of rural regions and cities across a large area of a federal state and (ii) covers a spectrum of different mental disorders. This approach offered the opportunity of representing a large variety of housing support forms and its different users.

Enfacing the number of persons in need of supported housing, economic considerations [26], and most of all the users’ preferences [27], our main results implicate important considerations regarding mental health care policy and access to housing services for persons with a severe mental illness. As there appeared overall positive psychosocial and clinical outcomes regardless of the supported housing type, one can conclude that supported accommodation per se appears to be an effective rehabilitative care approach (compare to [1]). Since no randomization was carried out, this could imply on one hand, that the participants in our study had received exactly the right housing type for themselves through the preceding rehabilitation planning. However, considering our matching procedure leading to two very well-parallelized study groups in terms of social functioning, quality of life, psychopathology and sociodemographics, our results might also indicate that it might not play a major role, in which form of housing a person is supported. At least, such an interpretation might be true for those persons concerned, who are willing to participate in a research project. Moreover, whether this assumption can be confirmed under rigorous scientific procedures will be clarified by an ongoing controlled study in which clients are indeed randomly assigned to the different housing conditions [28].

All in all, the present findings seem to support the expansion of supported housing programs and ensuring the availability of affordable housing for people with mental health problems. This would not only be a reasonable future perspective under financial aspects [26, 29, 30], but also by taking the users’ needs and wishes even more into account: a recent review and meta-analysis on the choice of housing in people with mental disorders revealed that 84% of the persons with severe mental illness preferred to live under more independent housing settings [27]. The importance of user preferences should not be underestimated, because studies have shown the positive effects of choice on psychosocial outcomes in mental health supported housing [31, 32].

### Study group characteristics

In light of the real-life study conditions, the data from our initial ITT sample additionally offer some descriptive insights into the client-structure of the RC and SH care, although it is of course not a complete epidemiologic survey. For instance, there were about twice as many men as women in the residential housing group, while the proportion was more balanced under independent housing conditions. However, this finding is largely consistent with both previous data from our region [33] and studies from neighboring European countries [34–37]. Moreover, such gender differences are also very likely related to diagnosis differences, because it is considered proven that woman had significantly higher risk of mood disorders than men, and men had more substance disorders and a higher incidence of schizophrenia [38, 39]. Thus, in fact, there was a significant difference in the distribution of psychiatric disorders in our ITT sample, which is broadly consistent with the findings of previous studies (e.g., [34, 35]): while individuals with mood (and anxiety) disorders are more frequently found in more independent housing settings than in residential care, the opposite pattern seems to be true for substance use and schizophrenic disorders. That is, residential housing services are more often used by individuals with schizophrenia (or substance use) disorders rather than affective disorders. Moreover, in a large register-based study, it was found, that the majority of persons who became residents in supported psychiatric housing facilities had previously been diagnosed with schizophrenia, schizophrenia-like disorders, and organic mental disorders, and a large proportion had substance abuse [37]. Furthermore, this study had identified schizophrenia as the strongest diagnostic predictor of becoming a resident in a supported psychiatric housing facility [37]. However, a significant majority of individuals with severe mental illness, including many with schizophrenic disorders, actually prefer independent supported living rather than institutionalized housing settings [27].

### Limitations

Although our study contributes to fill a research gap and addresses the need for more empirical studies on supportive housing for non-homeless people with mental disorders, some limitations of the present study should be considered. First of all, due to ethical and practical barriers (see Introduction), our study does not fulfil the highest methodological research quality associated with a randomized controlled trial (RCT) design. However, this is a generally known challenge for housing research [5, 6], because “*the RCT is not immune to problems common in community trials*” (p. 1363) [40]. Furthermore, in a Cochrane review, Anglemyer et al. (2014) found that there is only little evidence for significant differences between observational studies and RCTs in the field. In the present study, we followed a rigorous statistical approach to ensure the greatest possible validity. Especially, analyses using a propensity score matched sample is known to mimic that of an RCT [41]. However, our missing-value approach using the Last-Observation-Carried-Forward (LOCF) method must be considered critically. Although recent evaluations show that this method is still frequently used (even if misinterpreted as a conservative approach), its susceptibility to biases actually caused it to be called one of the “antiquated techniques” or “most egregious methods” [42]. In the present study, however, the results could be corroborated by the use of the advanced expectation maximization (EM) missing-value procedure. Another limitation is that of those initially eligible, only 49.4% of the persons (*n *= 257) agreed to participate in the study. One possible explanation for this could be that those who agreed to participate were in a better (mental) health condition than those who did not. However, for data protection reasons, it was not possible to compare relevant clinical or sociodemographic variables of nonparticipants with those of the study clients. Moreover, the present study is still deficient in that the participants ICD-10 diagnoses solely stem from the available medical documentation and were not re-validated through a standardized diagnostic assessment or interview as the gold standard in clinical research. On one hand, this might compromise the internal validity of our study, but on the other hand, it strengthens the representation of the naturalistic conditions of residential housing service settings. This is, we did not study a highly selected, standardized sample, but included people who use the housing services under typical real-life community conditions.

### Conclusion

To sum up our main findings, in consideration of the aforementioned limitations, the present study could not identify any differences between Supported Housing and Residential Care for non-homeless people with severe mental illness regarding different clinical and psychosocial outcomes. Thus, by also taking into account the users' general preferences, the present results therefore give rise to support endeavours of a further expansion of more independent supported housing approaches.

## Supplementary Information

Below is the link to the electronic supplementary material.

Supplementary file1 (DOCX 33 KB)

## Data Availability

The data that support the findings of this study are available from the corresponding author upon reasonable request.
