# Supplementary material for: Effectiveness of supported housing versus residential care in severe mental illness: a multicenter, quasi-experimental study
Source: Soc Psychiatry Psychiatr Epidemiol. 2022 Jan 18;57(5):927–37. doi: 10.1007/s00127-021-02214-6 (PMC9042980; doi:10.1007/s00127-021-02214-6)
Supplement: Supplementary file 1 — Supplementary file1 (DOCX 33 KB) [file 127_2021_2214_MOESM1_ESM.docx]

**Supplemental Material**

Effectiveness of Independent Housing and Support versus Residential Care in Severe Mental Illness: A Multicenter, Quasi-Experimental Follow-up Study

**Table S1**. Demographic and clinical baseline characteristics of the propensity-score-matched per-protocol sample [M(SD), n(%)]

|  | **Propensity score matched**  **Per-Protocol sample** | | | |
| --- | --- | --- | --- | --- |
|  | **RC**  (n=51) | **IHS**  (n=51) | **Total**  (N=102) | **Statistic**  (RC vs. IHS) |
| Sex (female) | 18 (35.3) | 23 (45.1) | 41  (40.2) | Chi^2^(1)=1.02, *p*=.419,  V=.100 |
| Age | 43.2 (14.8) | 43.0 (12.6) | 43.1 (13.7) | T(97.5)=0.65, *p*=.948,  d=.015 |
| ICD Diagnosis |  |  |  |  |
| F1 | 18 (35.3) | 15 (29.4) | 33 (32.4) | Chi^2^(4)=1.72, *p*=.800,  V=.130 |
| F2 | 17 (33.3) | 14 (27.5) | 31 (30.4) |  |
| F3 | 5 (9.8) | 8 (15.7) | 13 (12.7) |  |
| F4 | 4 (7.8) | 6 (11.8) | 10 (9.8) |  |
| Other | 8 (13.7) | 8 (15.7) | 15 (14.7) |  |
| Prevalence of psychiatric admissions (12 months) | 35 (68.6) | 29 (56.9) | 64 (62.7) | Chi^2^(1)=1.5, *p*=.306,  V=.122 |
| SCL-9-K ^a^ | 11.2 (8.1) | 12.6 (8.5) | 11.9 (8.2) | T(100)=-.876, *p*=.383,  d=.169 |
| SFS total ^a^ | 104.9 (7.7) | 105.1 (8.9) | 105.0 (8.3) | T(100)=-.161 *p*=.872,  d=.024 |
| MANSA ^a^ | 38.6 (12.0) | 41.1 (15.0) | 39.9 (13.5) | T(100)=-.920, *p*=.360,  d=.184 |

^a^ based on the Last-Observation-Carried-Forward (LOCF) missing values handling method

**Table S2.** Results of the repeated measures ANOVAs for the propensity-score-matched (PSM) Per-protocol (P-P) sample (n=102) with LOCF- and EM-missing values handling method.

| Outcome | **PSM Per-Protocol sample**  **with LOCF-method**^a^ | | | **PSM Per-Protocol sample**  **with EM-method**^b^ | | |
| --- | --- | --- | --- | --- | --- | --- |
|  | Effects of time | Effects of group | Interaction  time x group | Effects of time | Effects of group | Interaction  time x group |
| Social Functioning (SFS total) | F=7.18, ***p*=.009**, d=.536 | F=0.68, *p*=.676, d=.089 | F=2.17, p=.144, d=.293 | F=7.15, ***p*=.009**,  d=.536 | F=0.74, *p*=.786,  d=.063 | F=1.96, *p*=.165,  d=.278 |
| Symptom Level  (SCL-K9) | F=6.10, ***p*=.015**, d=.496 | F=2.95, *p*=.089, d=.346 | F=2.90, *p*=.097, d=.333 | F=6.69, ***p*=.011,**  d=.519 | F=3.04, *p*=.084,  d=.352 | F=2.69, *p*=.104,  d=.327 |
| Quality of Life (MANSA) | F=12.74, ***p*=.001**, d=.714 | F=0.29, *p*=.593, d=.110 | F=1.22, *p*=.271, d=.220 | F=13.71, ***p*<.001**,  d=.742 | F=0.24, *p*=.625,  d=.090 | F=1.01, *p*=.317,  d=.201 |

^a^ LOCF = Last Observation Carried Forward

^b^ EM = maximum likelihood-based Expectation Maximization

Notes: df=1 in outcome variables, error df=100

**Table S3.** Result for the comparison of the occurrence of psychiatric hospitalisations in the last 12 months using the propensity-score-matched Per-protocol sample with LOCF-approach

|  | **RC (n=51)** | **IHS (n=51)** |
| --- | --- | --- |
| T1 🡪 T3 | 68.6% 🡪 13.7%,  McNemar-Test: **p<.001**, V=.147 | 56.9% 🡪 27.5%,  McNemar-Test: **p=.001**, V=.358 |
| T1 | Chi^2^(1)=1.51, p=.306, V=.122 | |
| T3 | Chi^2^(1)=2.94, p=.141, V=.170 | |

**Table S4**. Results of the repeated measures ANOVAs for the total Intention-to-treat (ITT) sample (n=210) with LOCF and EM missing values handling method (RC: n=83, IHS: n=127)

| Outcome | **Intention-to-treat sample**  **with *LOCF*-method^a^** | | | **Intention-to-treat sample**  **with *EM-*method^b^** | | |
| --- | --- | --- | --- | --- | --- | --- |
|  | Effects of time | Effects of group | Interaction  time x group | Effects of time | Effects of group | Interaction  time x group |
| Social Functioning (SFS total) | F=24.63, ***p*<.001**,  d=.689 | F=0.93, *p*=.336,  d=.127 | F=3.27, *p*=.072,  d=.247 | F=28.05, ***p*<.001**,  d=.735 | F=1.28, *p*=.260,  d=.155 | F=2.78, *p*=.097,  d=.230 |
| Symptom Level  (SCL-K9) | F=14.73, ***p*=.001,**  d=.532 | F=7.14, ***p*=.008**,  d=.370 | F=0.86, *p*=.354,  d=.127 | F=18.47, ***p*<.001,**  d=.598 | F=7.04, ***p*=.009**,  d=.370 | F=0.56, *p*=.454,  d=.110 |
| Quality of Life (MANSA) | F=28.18, ***p*<.001**,  d=.735 | F=0.23, *p*=.632,  d=.063 | F=0.17, *p*=.682,  d=.063 | F=47.56, ***p*<.001**,  d=.956 | F=0.74, *p*=.390,  d=.127 | F=1.19, *p*=.277,  d=.155 |
| ^a^ LOCF = Last Observation Carried Forward  ^b^ EM = maximum likelihood-based Expectation Maximization  Notes: df=1 in outcome variables, error df=163 | | | | | | |

**Table S5.** Result for the comparison of the occurrence of psychiatric hospitalisations in the last 12 months using the total Intention-to-treat (ITT) sample with LOCF

|  | **RC (n=61)** | **IHS (n=104)** |
| --- | --- | --- |
| T1 🡪 T3 | 73.8% 🡪 19.7%,  McNemar-Test: **p<.001**, V=.201 | 45.2% 🡪 20.2%,  McNemar-Test: **p<.001**, V=.313 |
| T1 | Chi^2^(1)=12.73, **p<.001**, V=.278 | |
| T3 | Chi^2^(1)=0.01, p=1.00, V=.006 | |

**Table S6**. Descriptive data for the numerical outcomes of each subsample [M(SD)]

| **Sample** ^a^ |  | **RC** | | | **IHS** | | |
| --- | --- | --- | --- | --- | --- | --- | --- |
| PSM ITT sample  (LOCF-method)  [N=124] | T1 SFS | 104.8 (9.2) | n=62 | 104.9 (8.4) | | n=62 |  |
|  | T3 SFS | 107.5 (9.4) |  | 106.0 (9.2) | |  |  |
|  | T1 SCL-K9 | 11.5 (8.1) |  | 13.1 (8.5) | |  |  |
|  | T3 SCL-K9 | 9.7 (7.2) |  | 12.7 (8.5) | |  |  |
|  | T1 MANSA | 38.0 (11.8) |  | 38.5 (15.7) | |  |  |
|  | T3 MANSA | 41.9 (14.4) |  | 42.5 (14.2) | |  |  |
| PSM ITT sample  (EM-method)  [N=124] | T1 SFS | 105.1 (9.0) | n=62 | 104.8 (8.5) | | n=62 |  |
|  | T3 SFS | 107.9 (8.5) |  | 106.1 (8.7) | |  |  |
|  | T1 SCL-K9 | 11.4 (8.0) |  | 13.2 (8.5) | |  |  |
|  | T3 SCL-K9 | 9.6 (6.6) |  | 12.7 (8.5) | |  |  |
|  | T1 MANSA | 38.2 (11.7) |  | 38.4 (15.7) | |  |  |
|  | T3 MANSA | 43.7 (12.5) |  | 42.3 (13.8) | |  |  |
| Total  unmatched  ITT sample  (LOCF-method)  [N=210] | T1 SFS | 105.1 (8.8) | n=83 | 104.9 (8.4) | | n=127 |  |
|  | T3 SFS | 108.2 (8.8) |  | 106.3 (8.2) | |  |  |
|  | T1 SCL-K9 | 12.3 (8.1) |  | 14.7 (8.7) | |  |  |
|  | T3 SCL-K9 | 10.0 (7.4) |  | 13.3 (8.7) | |  |  |
|  | T1 MANSA | 37.4 (11.1) |  | 36.9 (15.4) | |  |  |
|  | T3 MANSA | 41.9 (13.5) |  | 40.7 (13.8) | |  |  |
| Total  unmatched  ITT sample  (EM-method)  [N=210] | T1 SFS | 105.3 (8.6) | n=83 | 104.8 (8.4) | | n=127 |  |
|  | T3 SFS | 108.3 (8.0) |  | 106.4 (7.9) | |  |  |
|  | T1 SCL-K9 | 12.3 (8.1) |  | 14.8 (8.6) | |  |  |
|  | T3 SCL-K9 | 10.1 (6.8) |  | 13.3 (8.5) | |  |  |
|  | T1 MANSA | 37.6 (11.0) |  | 36.8 (15.4) | |  |  |
|  | T3 MANSA | 43.0 (11.6) |  | 40.7 (13.5) | |  |  |
| PSM  P-P sample  (LOCF-method)  [N=102] | T1 SFS | 104.9 (7.7) | n=51 | 105.2 (8.9) | | n=51 |  |
|  | T3 SFS | 107.5 (8.4) |  | 105.9 (9.3) | |  |  |
|  | T1 SCL-K9 | 11.2 (8.1) |  | 12.6 (8.5) | |  |  |
|  | T3 SCL-K9 | 8.6 (6.9) |  | 12.1 (8.3) | |  |  |
|  | T1 MANSA | 38.7 (12.0) |  | 41.1 (14.9) | |  |  |
|  | T3 MANSA | 43.5 (13.9) |  | 43.7 (13.1) | |  |  |
| PSM  P-P sample  (EM-method)  [N=102] | T1 SFS | 104.8 (7.7) | n=51 | 105.2 (8.9) | | n=51 |  |
|  | T3 SFS | 107.4 (8.4) |  | 106.0 (9.3) | |  |  |
|  | T1 SCL-K9 | 11.2 (8.1) |  | 12.7 (8.5) | |  |  |
|  | T3 SCL-K9 | 8.5 (6.9) |  | 12.1 (8.3) | |  |  |
|  | T1 MANSA | 38.7 (12.0) |  | 41.0 (15.0) | |  |  |
|  | T3 MANSA | 43.5 (13.9) |  | 43.7 (13.1) | |  |  |
| ^a^ Abbreviations: *PSM = Propensity Score Matching \| ITT = Intention to Treat \| LOCF = Last Observation Carried Forward \| EM = maximum likelihood-based Expectation Maximization \| P-P = Per-Protocol* | | | | | | |  |
